# Putative hexameric glycosyltransferase functional unit revealed by the crystal structure of *Acinetobacter baumannii* MurG

**DOI:** 10.1107/S2052252521003729

**Published:** 2021-05-08

**Authors:** Kyoung Ho Jung, Sunghark Kwon, Chang Min Kim, Jun Hyuck Lee, Hyun Ho Park

**Affiliations:** a College of Pharmacy, Chung-Ang University, Seoul 06974, Republic of Korea; b Department of Biotechnology, Konkuk University, Chungju, Chungbuk 27478, Republic of Korea; c Research Unit of Cryogenic Novel Material, Korea Polar Research Institute, Incheon 21990, Republic of Korea; d Department of Polar Sciences, University of Science and Technology, Incheon 21990, Republic of Korea

**Keywords:** *Acinetobacter baumannii*, cell-wall peptidoglycan biosynthesis, crystal structure, glycosyltransferases, MurG, superbugs

## Abstract

Lipid II, the main component of the bacterial cell wall, is synthesized by the addition of UDP-*N*-acetylglucosamine to the UDP-*N*-acetylmuramic acid pentapeptide catalyzed by the glycosyltransferase MurG. Owing to its critical role in cell-wall biosynthesis, MurG is considered to be an attractive target for antibacterial agents. Although the Mur family ligases have been extensively studied, the molecular mechanism of the oligomeric scaffolding assembly of MurG remains unclear. In this study, MurG from *Acinetobacter baumannii* (abMurG), a human pathogen, was characterized and its hexameric crystal structure was unveiled; this is the first homo-oligomeric structure to be described in the MurG family and the Mur family. Homogeneous protein samples were produced for structural studies using size-exclusion chromatography, the absolute molecular mass was calculated via multi-angle light scattering, and protein–protein interactions were analyzed using the *PDBePISA* server. abMurG was found to form homo-oligomeric complexes in solution, which might serve as functional units for the scaffolding activity of MurG. Furthermore, analysis of this structure revealed the molecular assembly mechanism of MurG. This structural and biochemical study elucidated the homo-oligomerization mechanism of MurG and suggests a new potential antibiotic target on MurG.

## Abstract 2

The glycosyltransferase MurG from the human pathogen *A. baumannii* was characterized and its hexameric crystal structure was unveiled. This is the first homo-oligomeric structure to be described in the MurG family and the Mur family. The homo-oligomerization mechanism of MurG is clarified, thus opening the door for the design of next-generation antibiotics that target MurG.

## 1. Introduction   

Peptidoglycan, the major component of the bacterial cell wall, is a three-dimensional mesh structure formed by the polymerization of repeating disaccharide subunits cross-linked by short peptides (Vollmer & Bertsche, 2008 ▸; Matteï *et al.*, 2010 ▸). As peptidoglycan is essential for the growth and division of the cell wall of all eubacteria, the peptidoglycan-synthesis pathway is an attractive target for antibacterial agents (Chung *et al.*, 2016 ▸; Basavannacharya *et al.*, 2010 ▸; Kouidmi *et al.*, 2014 ▸).

Peptidoglycan synthesis occurs in three different bacterial compartments: the cytoplasm, the membrane and the periplasm. During the cytoplasmic step, lipid II, the main peptidoglycan building unit, is produced by the action of the Mur family of enzymes (MurA–MurG; Smith, 2006 ▸; Miyachiro *et al.*, 2019 ▸). Initially, MurA and MurB catalyze the generation of UDP-*N*-acetylmuramic acid (MurNAc) from UDP-*N*-acetyl­glucosamine (GlcNAc). Subsequently, a peptide moiety is assembled on UDP-MurNAc by the successive addition of l-alanine, d-glutamine, diaminopimelic acid or l-lysine, and dipeptide d-alanyl-d-alanine, generating the UDP-MurNAc pentapeptide. These additions are catalyzed by the Mur ligases MurC, MurD, MurE and MurF, respectively.

**Figure 1:**
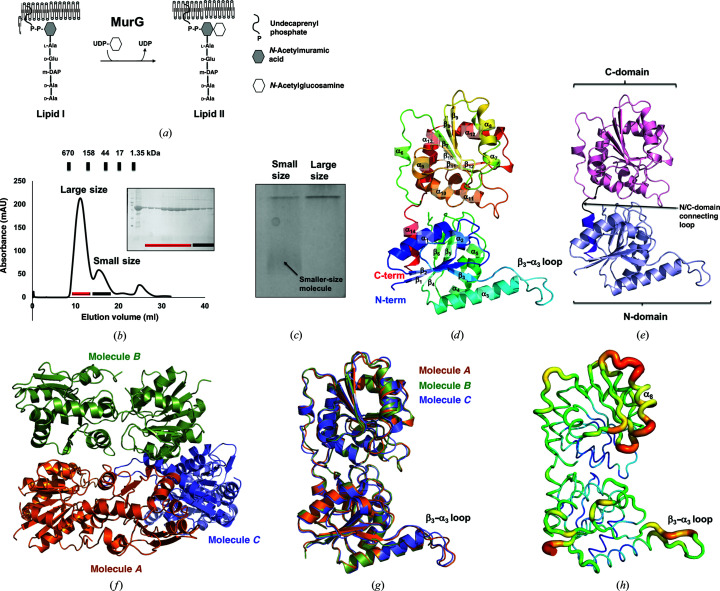
Crystal structure of abMurG. (*a*) Overview of the function of MurG. (*b*) Size-exclusion chromatography profile. Two peaks are labeled corresponding to an oligomer and a dimer. SDS–PAGE to assess the identity and purity is shown to the right of the two main peaks. The loaded fractions are indicated by black and red bars. (*c*) Native PAGE gel. The loaded samples are indicated above the gel. (*d*) Multi-colored cartoon representation of monomeric abMurG. The chain from the N- to C-terminus is colored from blue to red. Helices and sheets are labeled α and β, respectively. (*e*) A cartoon representation of the structure of abMurG showing the domain boundary in the structure. (*f*) Cartoon representation of the three abMurG molecules in an asymmetric unit. (*g*) Superposition of the structures of the molecules found in one asymmetric unit. (*h*) Putty representation showing the *B*-factor distribution. Rainbow colors from red to violet with increasing *B*-factor values were used for *B*-factor visualization.

In contrast, MurG belongs to the glycosyltransferases, which are one of the most diverse enzymatic groups. It binds to phospholipids on the cytoplasmic side of the membrane (Bupp & Van Heijenoort, 1993 ▸). Further, the N-terminal hydrophobic patch of MurG is involved in membrane attachment (Ha *et al.*, 2000 ▸). This enzyme catalyzes the addition of UDP-GlcNAc (another disaccharide subunit) to the UDP-MurNAc pentapeptide (called lipid I) at the end of the cytoplasmic stage, producing lipid II, which is then moved to the periplasm by flippases for further modification, polymerization and cross-linking to generate peptidoglycan [Fig. 1 ▸(*a*)] (Sham *et al.*, 2014 ▸).

Considering that the enzymes involved in the peptido­glycan-biosynthesis pathway, including those belonging to the Mur family, are essential for cell viability, they are considered to be promising targets for antibacterial agents (Kouidmi *et al.*, 2014 ▸). Although many clinically used antibiotics, such as penicillin derivatives (β-lactams), vancomycin (glycopeptide) and cycloserine, target this peptidoglycan-biosynthesis pathway and have shown effective interference, no antibiotics targeting the Mur family are commercially available.

The need for the development of new antibiotic agents has recently been emphasized because of the emergence of superbugs, which are antibiotic-resistant bacteria that are considered to be one of the greatest threats to global public health (Kumar, 2016 ▸; Burki, 2018 ▸). For example, *Acinetobacter baumannii*, a typical rod-shaped Gram-negative bacterium, is a human pathogen that causes hospital-derived infections and occasionally exhibits multiple drug resistance.

Owing to the critical activity of MurG in bacterial cell-wall synthesis and its attractiveness as an antibiotic target, structural and biochemical studies have been performed in order to understand its molecular mechanism (Ha *et al.*, 1999 ▸; Mohammadi *et al.*, 2007 ▸; van den Brink-van der Laan *et al.*, 2003 ▸; Hu *et al.*, 2004 ▸). Although previous studies of *Escherichia coli* MurG (ecMurG) have shown how MurG functions in the membrane and how its active site recognizes substrates (Ha *et al.*, 2000 ▸; Hu *et al.*, 2003 ▸), the molecular mechanism of the oligomeric scaffolding assembly of MurG, as identified in several bacterial species in recent studies (Laddomada *et al.*, 2019 ▸), has remained unclear. This study characterized MurG from *A. baumannii* (abMurG). We revealed that abMurG forms a homo-oligomeric complex in solution that might be the functional unit for MurG scaffolding activity. We also determined the hexameric crystal structure of abMurG, which, to the best of our knowledge, is the first homo-oligomeric structure in the MurG family and even in the Mur family. Our structural study of hexameric MurG revealed the molecular assembly mechanism of MurG. These structural and biochemical studies are expected to shed light on the homo-oligomerization mechanism of MurG and to provide critical structural information for the design of drugs targeting MurG.

## 2. Materials and methods   

### 2.1. Protein expression and purification   

The expression plasmid for full-length abMurG corresponding to amino acids Met1–Met365 was constructed by inserting the synthesized gene product, digested at the NdeI and XhoI restriction sites, into pET-21a vector. The gene sequence was derived from GenBank (ID SVJ97884), and gene synthesis was conducted by BIONICS (Seoul, Republic of Korea). The expression vector containing the abMurG gene was delivered into the *E. coli* BL21 (DE3) strain by heat shock at 42°C. The transformed bacteria were spread on a lysogeny broth agar plate containing kanamycin and incubated at 37°C for 16 h. A single recombinant colony was selected and cultured overnight at 37°C in 5 ml lysogeny broth containing 50 µg ml^−1^ kanamycin, following which the cells were transferred and cultured on a large scale (6 l). When the optical density at 600 nm reached approximately 0.6–0.7, 0.5 m*M* isopropyl β-d-1-thiogalactopyranoside was added to the medium to induce gene expression, and the cells were further cultured at 20°C for 18 h in a shaking incubator. Subsequently, the bacterial cells were harvested by centrifugation and the pellet was resuspended in 20 ml lysis buffer (20 m*M* Tris–HCl pH 7.9, 500 m*M* NaCl). After adding phenylmethylsulfonyl fluoride, a serine protease inhibitor (Sigma–Aldrich, St Louis, USA), the cells were disrupted by sonication on ice with six bursts of 30 s each and a 60 s interval between bursts. The cell lysate was centrifuged at 10 000*g* and 4°C for 30 min to remove cell debris. The supernatant was collected and mixed with nickel–nitrilotriacetic acid resin solution (Qiagen, Hilden, Germany) by gentle agitation at 4°C overnight. The resulting mixture was inserted into a gravity-flow column pre-equilibrated with lysis buffer. The column was washed with 200 ml washing buffer (20 m*M* Tris–HCl pH 7.9, 500 m*M* NaCl, 25 m*M* imidazole) to remove unbound proteins. 3 ml elution buffer (20 m*M* Tris–HCl pH 7.9, 500 m*M* NaCl, 250 m*M* imidazole) was then loaded into the column to elute the bound protein. The resulting eluate was concentrated to 20 mg ml^−1^ and was subsequently subjected to size-exclusion chromatography (SEC). SEC purification was conducted using an ÄKTAexplorer system (GE Healthcare, Chicago, USA) equipped with a Superdex 200 Increase 10/300 GL 24 ml column (GE Healthcare) pre-equilibrated with SEC buffer (20 m*M* Tris–HCl pH 8.0, 150 m*M* NaCl). The SEC peak fractions were pooled, concentrated to 8 mg ml^−1^, flash-cooled in liquid N_2_ and stored at −80°C until use. Protein purity was assessed by SDS–PAGE.

### 2.2. SEC-MALS analysis   

The absolute molar mass of full-length abMurG in solution was determined using multi-angle light scattering (MALS). The target protein, purified by affinity chromatography using nickel–nitrilotriacetic acid resin, was filtered using a 0.2 µm syringe filter and loaded onto a Superdex 200 10/300 gel-filtration column (GE Healthcare) that had been pre-equilibrated with SEC buffer. The mobile phase buffer was flowed at a rate of 0.4 ml min^−1^ at 25°C. A DAWN TREOS MALS detector (Wyatt Technology, Santa Barbara, USA) was connected to the ÄKTAexplorer system (GE Healthcare). The molecular mass of bovine serum albumin was used as the reference value. The absolute molecular mass was assessed using *ASTRA* (Wyatt Technology).

### 2.3. Native PAGE   

Different oligomeric states of abMurG in solution were assayed by native (nondenaturing) PAGE conducted on a PhastSystem (GE Healthcare) with pre-cast 8–25% acrylamide gradient gels (GE Healthcare). Coomassie Brilliant Blue was used for the staining and detection of bands. Different oligomeric states were evaluated based on the appearance of lower and upper bands on the native PAGE.

### 2.4. Crystallization and data collection   

For initial crystal screening, 1 µl 8 mg ml^−1^ protein solution in 20 m*M* Tris–HCl pH 8.0, 150 m*M* NaCl was mixed with an equal volume of reservoir solution and the droplet was allowed to equilibrate against 500 µl mother liquor using the hanging-drop vapor-diffusion method at 20°C. Crystals were initially obtained using a buffer consisting of 0.1 *M* Tris–HCl pH 8.0, 2.5 *M* NaCl, 0.2 *M* MgCl_2_. The crystallization conditions were further optimized and finally adjusted to a buffer composition of 0.1 *M* Tris–HCl pH 8.0, 2.6 *M* NaCl, 0.15 *M* calcium acetate. Diffraction-quality crystals appeared in three days and grew to maximum dimensions of 0.1 × 0.1 × 0.4 mm. For data collection, the crystals were soaked in mother liquor supplemented with 30%(*v*/*v*) glycerol as a cryoprotectant, mounted and flash-cooled in an N_2_ stream at −178°C. The diffraction data were collected on the 5C beamline at the Pohang Accelerator Laboratory (PAL), Pohang, Republic of Korea at a wavelength of 0.9735 Å. The diffraction data were indexed, integrated and scaled using *HKL*-2000 (Otwinowski & Minor, 1997 ▸).

### 2.5. Structure determination and analysis   

The abMurG structure was determined by molecular replacement using *Phaser* (McCoy, 2007 ▸). The ecMurG structure (PDB entry 1f0k; Ha *et al.*, 2000 ▸), which has 43% amino-acid sequence homology to abMurG, was used as the search model. The initial model was built automatically with *AutoBuild* in *Phenix* (Liebschner *et al.*, 2019 ▸) and was completed with *Coot* (Emsley *et al.*, 2010 ▸). Model refinement was iteratively performed using *phenix.refine* in *Phenix*. The quality of the model was validated using *MolProbity* (Chen *et al.*, 2010 ▸). All structural figures were generated using *PyMOL* (DeLano & Lam, 2005 ▸).

### 2.6. Mutagenesis   

Site-directed mutagenesis was conducted using a QuikChange kit (Stratagene) according to the manufacturer’s protocols. Mutagenesis was then confirmed by sequencing. Mutant proteins were prepared using the method described above.

### 2.7. Sequence alignment   

The amino-acid sequences of MurG from various species were analyzed using *Clustal Omega* (http://www.ebi.ac.uk/Tools/msa/clustalo/).

### 2.8. Accession code   

Coordinates and structure factors have been deposited in the RCSB Protein Data Bank with PDB code 7d1i.

## 3. Results and discussion   

### 3.1. Overall structure of abMurG   

To explore the structure of abMurG, the full-length MurG cDNA from *A. baumannii*, coding for a protein of 365 amino acids, was synthesized and cloned into pET-21a expression vector. To produce homogeneous protein samples for structural studies, we conducted quick two-step chromatography: affinity chromatography followed by SEC. This purification process generated two homogeneous protein samples corresponding to the large-size and small-size peaks, which were used for crystallization [Fig. 1 ▸(*b*)]. Only protein sample from the large-size peak was successfully crystallized. On the native PAGE, a smear of putative smaller-sized molecules of MurG was detected, indicating that MurG forms both large and small oligomeric molecules in solution [Fig. 1 ▸(*c*)]. Finally, a 3.49 Å resolution crystal structure of abMurG was solved and refined to *R*_work_ = 22.28% and *R*_free_ = 27.16%. The crystallographic and refinement statistics are summarized in Table 1 ▸.

The crystal structure of abMurG showed the typical fold of MurG, containing two distinct domains (N- and C-domains) [Figs. 1 ▸(*d*) and 1 ▸(*e*)]. It was composed of 14 α-helices and 12 β-sheets, and there was an extraordinarily long loop between β3 and α3 (hereafter named the β3–α3 loop) [Fig. 1 ▸(*d*)]. The two distinctly separated N- and C-domains, which exhibit an α/β open-sheet structure, were connected by an N/C-domain connecting loop [Fig. 1 ▸(*e*)]. The N-domain consisted of six α-helices (α1–α5 and α14) and six β-sheets (β1–β6), whereas the C-domain consisted of eight α-helices (α6–α12) and six β-sheets (β7–β12) [Figs. 1 ▸(*d*) and 1 ▸(*e*)]. The last helix at the C-terminus, α14, was located in the N-domain; it was involved in forming the α/β open-sheet structure of the N-domain [Figs. 1 ▸(*d*) and 1 ▸(*e*)]. Three molecules were found in the asymmetric unit: *A*, *B* and *C* [Fig. 1 ▸(*f*)]. Models of each molecule were constructed and included residues 8–365 for molecules *A* and *B*, and residues 9–365 for molecule *C*. Seven residues from the N-terminus and several loops were not included in the model due to poor electron density. The final model contained residues 8–166 and 175–365 for molecule *A*, residues 8–66, 77–168 and 175–365 for molecule *B*, and residues 9–166, 173–295 and 299–365 for molecule *C* [Fig. 1 ▸(*f*)]. In the asymmetric unit, molecule *B* was stacked on molecule *A*, and molecule *C* was on the right side of molecule *A* [Fig. 1 ▸(*f*)]. The structures of the three molecules in the same asymmetric were nearly identical, with root-mean-square deviations of 0.65–0.87 Å over 338 C^α^ atoms [Fig. 1 ▸(*g*)]. Despite this structural similarity, structural discrepancy was detected in the region of the β3–α3 loop. The position of this loop in molecule *A* was not identical to that in molecule *C*. In molecule *B*, this loop was not included in the final model due to untraceable electron density, indicating that this region might be flexible. *B*-factor analysis showed that the structure of abMurG contained two high *B*-factor regions, including the β3–α3 loop and α8 and its connecting loops [Fig. 1 ▸(*h*)], which supported our hypothesis that the β3–α3 loop is a flexible region in the structure of abMurG.

### 3.2. abMurG forms hexameric homo-oligomeric complex structures   

**Figure 2:**
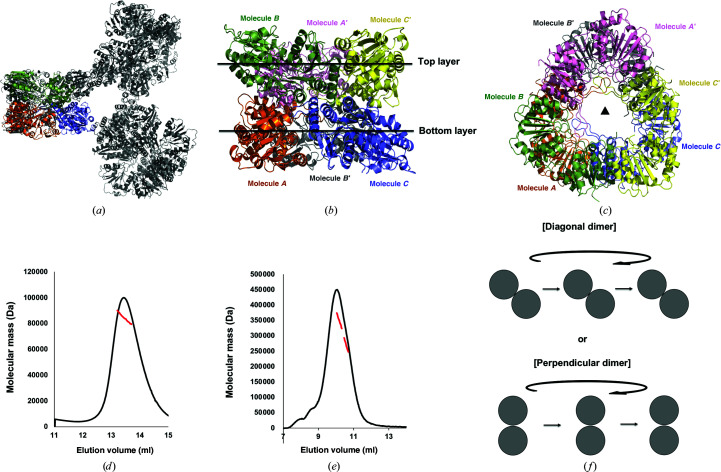
Hexameric structure of abMurG. (*a*) Crystallographic packing symmetry analysis. Three abMurG molecules in the asymmetric unit are indicated by color cartoons, whereas other symmetric molecules are indicated by gray ribbon structures. (*b*, *c*) Tentative hexameric structure of abMurG generated by the symmetry analysis: side (*b*) and top (*c*) views. (*d*, *e*) Multi-angle light scattering (MALS) profiles derived from the first (*d*) and second (*e*) size-exclusion chromatography peaks. The red line indicates the experimental molecular mass analyzed by MALS. (*f*) Schematic planar diagram showing the hexamer assembly strategy of two tentative dimers.

Although structural studies have provided no direct evidence, a highly oligomeric state of MurG has consistently been proposed as a functional unit by biochemical, biophysical and cellular studies (Laddomada *et al.*, 2019 ▸; Ha *et al.*, 1999 ▸). As we solved the structure of abMurG from a highly oligomeric protein sample, we analyzed the crystallographic packing to search for symmetric molecules and found three molecules (*A*′, *B*′ and *C*′) that formed a hexameric structure with the three molecules found in the asymmetric unit [Fig. 2 ▸(*a*)]. This hexamer was formed by two trimeric molecules stacked against each other, forming a two-layer structure with threefold symmetry [Figs. 2 ▸(*b*) and 2 ▸(*c*)]. Molecules *A*′, *B* and *C*′ were located in the top layer and molecules *A*, *B*′ and *C* in the bottom layer [Fig. 2 ▸(*b*)]. The top view of the complex shows that the two trimeric layers did not completely overlap [Fig. 2 ▸(*c*)]. The bottom layer flipped, rotated approximately 30° and stacked on the bottom layer, indicating that the hexamer of abMurG is constructed of six identical subunits that are arranged as a trimer of asymmetric dimers with *D*3 (or 32) symmetry [Figs. 2 ▸(*b*) and 2 ▸(*c*)]. When the trimeric form of abMurG was considered as the basic unit for the formation of this hexameric complex, abMurG was first trimerized and two trimers then interacted to form the hexameric structure. Another possibility for hexamer formation is that the abMurG dimer might be a protomer of this hexameric complex, and the dimers further interact to extend and form the hexameric complex. In this case, the dimerized protomers of abMurG could be either diagonal or perpendicular dimers. To understand complex formation, we analyzed the exact stoichiometry of abMurG in solution by calculating the absolute molecular mass using MALS. We performed MALS experiments using the highly oligomeric and dimeric peaks obtained in SEC. The experimental molecular mass of the dimer-sized peak was 79.3 kDa (1.1% fitting error) [Fig. 2 ▸(*d*)]. The theoretical molecular weight of abMurG, including the C-terminal His tag, is 39.4 kDa; hence, this experimental value confirms that abMurG is a dimer in this peak. In contrast, the molecular mass of the highly oligomeric peak was 280.5 kDa (4.8% fitting error), confirming that abMurG is a hexamer in this peak [Fig. 2 ▸(*e*)]. Based on these results, we concluded that abMurG forms dimers, and not trimers, in solution, and that these dimers can further assemble into a highly oligomeric form; in this case, a hexamer. The dimeric central building block might be formed by diagonal or perpendicular dimers [Fig. 2 ▸(*f*)]. As both dimers might be formed in solution, furthur studies are needed to fully understand the assembly mechanism of hexameric abMurG.

**Figure 3:**
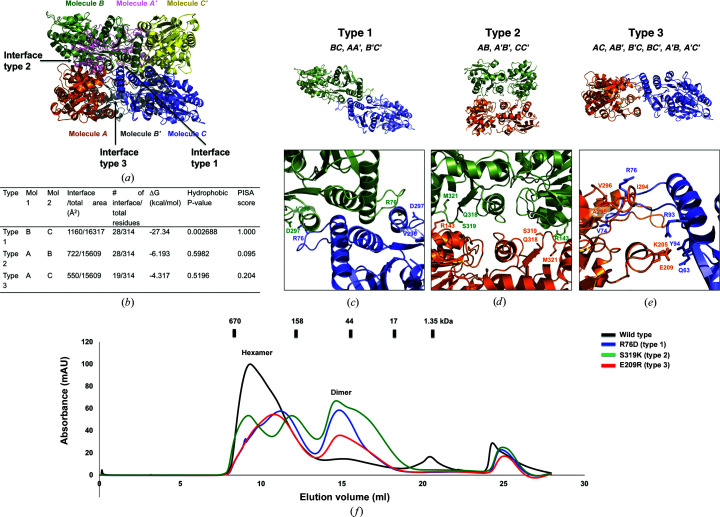
Details of the interface formed by the hexameric homo-oligomeric complex of abMurG. (*a*) Cartoon representation of the hexameric abMurG complex. Three different types of interfaces (types 1–3) formed by the hexameric complex are indicated by black arrows. (*b*) Table summarizing the interaction details of each type of interface. (*c*, *d*, *e*) Cartoon representation of each type of interaction interface: type 1 (*c*), type 2 (*d*) and type 3 (*e*). The position of each interface and the molecules involved in the formation of each interface are shown in the upper panel. Close-up views of each interface showing the residues involved in the formation of the interface are provided in the lower panels. (*f*) Verification of the interfaces by mutagenesis. Size-exclusion chromatography profiles comparing the positions of eluted peaks between wild-type abMurG and various mutants with disrupted type 1 (R76D), type 2 (S319K) and type 3 (E209R) interfaces.

To understand the assembly details of hexameric abMurG, we analyzed protein–protein interactions (PPIs) using *PDBePISA* (Krissinel & Henrick, 2007 ▸). According to the PPI computations, a hexameric quaternary structure was suggested as a stable form for abMurG in solution. The buried surface of the trimeric complex represented by the asymmetric unit was 5138 Å^2^ of the total accessible surface area of 44 718 Å^2^, which indicated that 11.4% of the surface was buried on the formation of a trimeric complex. When abMurG formed a hexameric complex, the total hexameric surface was 81 159 Å^2^ and the surface buried on formation of the hexameric complex was 18 554 Å^2^, representing 41.4% of the total surface area. Further analysis indicated that hexameric abMurG was formed using three different interactions (types 1, 2 and 3) [Fig. 3 ▸(*a*)]. If the three molecules (*A*, *B* and *C*) represented by the asymmetric unit are considered as building blocks for the hexameric structure, molecules *B* and *C* have type 1 inter­actions, molecules *A* and *B* have type 2 interactions, and molecules *A* and *C* have type 3 interactions [Figs. 3 ▸(*a*) and 3 ▸(*b*)]. According to the *PDBePISA* analysis, the interface of type 1 interactions had a complex-formation significance score of 1.000 (the score ranges from 0 to 1 as the relevance of the interface to complex formation increases), whereas the interfaces of type 2 and type 3 interactions had scores of 0.095 and 0.204, respectively [Fig. 3 ▸(*b*)]. These results imply that type 1 interactions are the most significant interaction force in forming the hexameric abMurG complex.

A total of 12 interfaces are formed using the three different types of interactions in hexameric abMurG: the inter­actions between molecules *B* and *C*, molecules *A* and *A*′, and molecules *B*′ and *C*′ are type 1 interactions [Fig. 3 ▸(*c*)], those between molecules *A* and *B*, molecules *A*′ and *B*′, and molecules *C* and *C*′ are type 2 interactions [Fig. 3 ▸(*d*)], and six interfaces, between molecules *A* and *C*, *A *and *B*′, *B*′ and *C*, *B* and *C*′, *A*′ and *B*, and *A*′ and *C*′, are formed by type 3 interactions [Fig. 3 ▸(*e*)]. Type 1 interactions mainly involve hydrogen bonds and salt bridges formed by the side chains of Arg76 and Asp297 and the main chain of Val296 [Fig. 3 ▸(*c*)]. Arg76 of one molecule forms salt bridges with Asp297 of the other molecule; Arg76 also forms hydrogen bonds to the main chain of Val296. In type 2 interactions, Arg143 forms hydrogen bonds to Gln318, Ser319 and Met321 from the neighboring molecule [Fig. 3 ▸(*d*)]. In the type 3 interface, Gln63, Val74, Arg76, Arg93 and Tyr94 of one molecule interact with Lys205, Glu209, Ile294, Ala295 and Val296 of the opposite molecule [Fig. 3 ▸(*e*)]. A structure-based mutagenesis study confirmed the analyzed interface. Arg76, Ser329 and Glu209 were analyzed as the main interface residues in type 1, type 2 and type 3 interactions, respectively. They were mutated to aspartic acid, lysine and arginine, producing the R76D, S319K and E209R mutants, respectively. Each mutant was purified, and the effects of the mutation in disruption of the hexameric complex were analyzed using SEC. The same protein concentration (∼2 mg ml^−1^) was used in the SEC experiments to compare the peak sizes. As indicated in Fig. 3 ▸(*f*), all three mutants had a definite disruptive effect on the hexameric complex, producing a higher dimeric peak than that produced by the wild type in the SEC profile. This indicates that abMurG forms a putative hexameric complex in solution, the assembly of which is mediated by the three types of inter­actions analyzed in this structural study. Notably, the S319K mutant produced one additional peak between the dimer and hexamer, implying that disruption of the type 2 interface by S319K mutagenesis produced new oligomeric forms.

### 3.3. Comparison of the abMurG and ecMurG structures   

**Figure 4:**
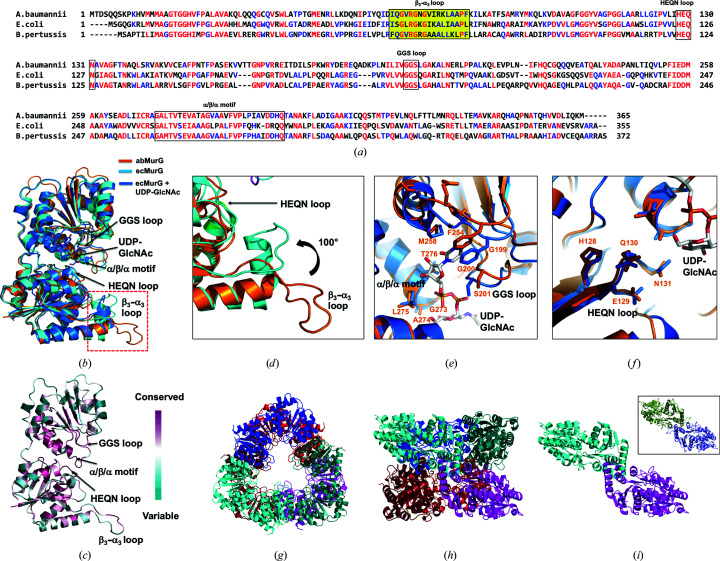
Structure and sequence comparison of abMurG and ecMurG. (*a*) Sequence alignment of MurGs from different species. Mostly conserved and partially conserved residues are shown in red and blue, respectively. The HEQN loop, GGS loop and α/β/α motif are indicated with black boxes. The position of the β3–α3 loop is highlighted. (*b*) Structural superposition of abMurG (orange) with ecMurG (cyan) and the ecMurG–UDP–GlcNAc complex (blue). The red dotted box indicates the β3–α3 loop region. (*c*) Cartoon representation of abMurG colored according to the degree of amino-acid sequence conservation. The HEQN loop, GGS loop and α/β/α motif regions, which are critical for the accommodation of substrates in the structure of abMurG, are indicated. (*d*) Different structural details of the β3–α3 loop region between abMurG (orange) and ecMurG (cyan). (*e*) Superimposition of the structure of abMurG with ecMurG focusing on the UDP–GlcNAc-binding site. (*f*) Superimposition of the structure of abMurG with ecMurG focusing on the HEQN loop. (*g*, *h*) Putative hexameric structure of ecMurG modeled using the hexameric abMurG structure as a template: top (*g*) and side (*h*) views. (*i*) Type 1 interaction between two molecules in the hexameric structure of ecMurG. The type 1 interaction in the hexameric structure of abMurG is shown in a black box for comparison.

To find evidence to infer the molecular mechanism underlying the hexameric assembly of abMurG and its functional role, we investigated its structural homologs using the *DALI* server (Holm & Sander, 1995 ▸) and compared each of these structures with that of abMurG. This investigation revealed two structures of ecMurG, a substrate-free form (PDB entry 1f0k; Ha *et al.*, 2000 ▸) and a UDP–GlcNAc-bound form (PDB entry 1nlm; Hu *et al.*, 2003 ▸), as the most structurally similar proteins (Table 2 ▸). Considering that most bacteria contain the MurG enzyme, it is noteworthy that ecMurG is the only structure reported to date; this might be because MurG is located on the membrane (van der Brink-van der Laan *et al.*, 2003 ▸), making its structural study challenging to perform because of solubility issues. Two other structures, MGD1 from *Arabidopsis* (PDB entry 4wyi; Rocha *et al.*, 2016 ▸) and KCN28 from *Kitasatospora* (PDB entry 6j31; Shi *et al.*, 2019 ▸), which are unrelated to MurG (with low sequence homology), were found as the third and fourth matches, respectively (Table 2 ▸). The sequence homology between abMurG and ecMurG was around 43%. ecMurG is the most enzymatically, biochemically and structurally studied MurG enzyme [Fig. 4 ▸(*a*)] (Men *et al.*, 1998 ▸; Ha *et al.*, 1999 ▸; Mohammadi *et al.*, 2007 ▸). Although previous studies indicate that *E. coli* and *Bordetella pertussis* MurG form an oligomeric scaffold during cell-wall synthesis (Laddomada *et al.*, 2019 ▸; Ha *et al.*, 1999 ▸), the structure of ecMurG has been found to be a monomer in the presence and absence of substrate (Ha *et al.*, 2000 ▸; Hu *et al.*, 2003 ▸). Structural comparison by the superposition of monomeric abMurG with monomeric ecMurG showed that the overall fold was the same, with a root-mean-square deviation of 1.5 Å. However, the positions and lengths of several loops in abMurG differed from those of the equivalent loops in ecMurG [Fig. 4 ▸(*b*)]. Nevertheless, the structures of the α/β/α motif and the GGS loop, which are essential for donor–substrate (UDP–GlcNAc) binding, and the HEQN loop, which is critical for accommodation of the acceptor substrate (lipid I), were conserved [Fig. 4 ▸(*b*)]. The sequences of these regions were also conserved among various species [Figs. 4 ▸(*a*) and 4 ▸(*c*)]. However, there were also regions with structural discrepancy. In particular, the β3–α3 loop of abMurG, which was predicted to be a flexible region, was in an extended form, whereas the β3–α3 loop of ecMurG was bent towards the HEQN loop [Figs. 4 ▸(*b*) and 4 ▸(*d*)]. This bending might be a critical structural transition to fix the acceptor substrate during enzymatic activity of MurG because the β3–α3 loop approaches the acceptor-binding site [Fig. 4 ▸(*d*)]. However, the bent β3–α3 loop in ecMurG was a common structural feature in the presence and absence of substrate [Fig. 4 ▸(*b*)] (Hu *et al.*, 2003 ▸; Ha *et al.*, 2000 ▸), and is possibly a structural feature of MurGs from several species, including *E. coli*. Structural comparison of the active site of abMurG with that of ecMurG showed that most residues around the active site were sequentially and structurally conserved. However, the precise locations of several residues, including Gly199, Gly200, Ser201 and Phe254, were not identical [Figs. 4 ▸(*e*) and 4 ▸(*f*)].

As it is known that ecMurG also forms a highly oligomeric complex in solution (Ha *et al.*, 1999 ▸), hexameric ecMurG was modeled using the hexameric abMurG structure as a template [Figs. 4 ▸(*g*) and 4 ▸(*h*)]. Because our abMurG structure indicated that the β3–α3 loop was involved in type 1 interactions and further assembly of the abMurG hexameric complex, we analyzed the role of this region of ecMurG in the proposed hexameric ecMurG complex. Analysis of the bent β3–α3 loop region in the hexameric structure of ecMurG showed that type 1 interactions failed because of a clash of the two molecules involved in these interactions, indicating that this bent loop inhibits MurG assembly [Fig. 4 ▸(*i*)].

### 3.4. Tentative working model of hexameric MurG on the membrane   

**Figure 5:**
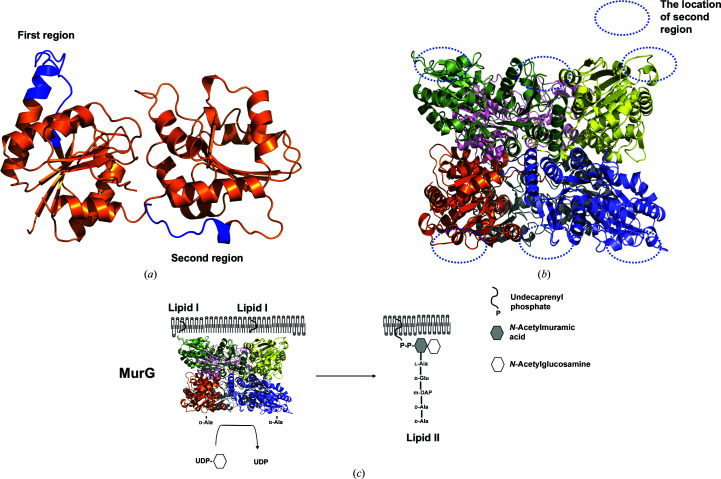
Proposed working model of hexameric MurG on the membrane. (*a*) Tentative membrane-docking regions on MurG. Membrane-anchoring regions were predicted by the *MODA* server. Two regions, colored blue, were analyzed as tentative membrane-docking regions. (*b*) The location of the second region, formed by a small helix connected by a loop, on each subunit of hexameric MurG. (*c*) Tentative working model of hexameric MurG on the membrane.

Based on previous reports and the findings of the current structural study, we concluded that MurG forms a putative hexameric complex, the assembly of which may be species-dependent. However, structural transition of the β3–α3 loop from the extended to the bent form might be critical for regulation of the hexameric complex in the hexamer-mediated scaffolding function of MurG, and this process might be common to all MurG enzymes. Because MurG is located on the membrane, it has been questioned how hexameric MurG can work on the membrane. In addition, a tentative model describing how a hexameric MurG would work with dimeric MraY and flippase components for feeding the substrate and flipping the product during lipid II formation, respectively, would be noteworthy. However, due to an absence of information on the membrane anchoring of hexameric MurG, it might be difficult to speculate on the membrane attachment of MurG to work on the lipidated substrate and its cooperativity with MraY and flippase on the membrane. To speculate on the tentative membrane-docking region in MurG, we used the *Membrane Protein Interface Recognition* (*MODA*) server (Kufareva *et al.*, 2014 ▸). According to the calculations by this server, two regions were picked as tentative membrane-docking regions [Fig. 5 ▸(*a*)]. One region was the β3–α3 loop part, which is critical for hexamer assembly. The other region was a small helix connected by a loop located on the surface of the MurG monomer [Fig. 5 ▸(*a*)]. This region was also exposed to the outside in hexameric MurG [Fig. 5 ▸(*b*)]. If the β3–α3 loop part is involved in hexamer formation, as analyzed in the current hexameric structure, the small helix connected by a loop located on the surface of MurG might be a candidate region for membrane anchoring. Based on this observation, we speculated on the working model of hexameric MurG in the intracellular part of the membrane [Fig. 5 ▸(*c*)]. MurG might wrap up the lipid I substrate on the membrane by forming a hexamer and anchoring to the membrane. In this state, *N*-acetyl­glucosamine (another substrate) might access the active site of hexameric MurG through the hole formed by subunits that are located on the opposite sides of membrane-anchoring subunits. Although a model has been proposed, the precise membrane-anchoring process and cooperativity mechanism of hexameric MurG with MraY and flippase need to be analyzed by either a structural study or a biochemical study.

Currently, the application of newly discovered MurG inhibitors as next-generation antibiotics stems from competitive substrate analogs, which are either UDP-GlcNAc-mimicking or lipid I-mimicking compounds. However, to date no successful results have been reported. If MurG oligomerization, as introduced in this study, is critical for the activity of this enzyme, targeting the interface might be an alternative approach. Overall, understanding the assembly mechanism may help in the design of next-generation antibiotics targeting MurG.

## Supplementary Material

PDB reference: MurG, 7d1i

## Figures and Tables

**Table 1:** Data-collection and refinement statistics Values in parentheses are for the outermost resolution shell.

Data collection	
Space group	*P*4_3_2_1_2
*a*, *b*, *c* (Å)	182.91, 182.91, 156.55
*α, β, γ* (°)	90, 90, 90
Resolution range (Å)	50.00–3.49
Total reflections	257452
Unique reflections	31262
Multiplicity	8.2 (3.4)
Completeness (%)	90.8 (82.1)
Mean *I*/σ(*I*)	10.2 (2.1)
*R*_merge_ † (%)	6.1 (44.1)
Wilson *B* factor (Å)	71.04
Refinement	
Resolution range (Å)	48.25–3.49
Reflections	31201
*R*_work_ (%)	22.28 (28.13)
*R*_free_ (%)	27.16 (33.28)
No. of molecules in the asymmetric unit	3
No. of non-H atoms	7706
Average *B*-factor values (Å)	
Molecule *A*	70.84
Molecule *B*	67.66
Molecule *C*	66.94
Ramachandran plot	
Favored (%)	96.42
Allowed (%)	3.58
Outliers (%)	0
Rotamer outliers (%)	4.2
Clashscore	6.23
Root-mean-square deviations	
Bonds (Å)	0.008
Angles (°)	0.928

† *R*_merge_ = \textstyle \sum_{hkl}\sum_{i}|I_{i}(hkl)- \langle I(hkl)\rangle|/\textstyle \sum_{hkl}\sum_{i}I_{i}(hkl), where *I*(*hkl*) is the observed intensity of reflection *hkl* and 〈*I*(*hkl*)〉 is the average intensity obtained from multiple measurements.

**Table 2:** Structural similarity search using *DALI*

Protein (PDB code)	*Z*-score	R.m.s.d. (Å)	Identity (%)	Reference
MurG from *E. coli* (1f0k)	42.9	1.5 over 331 C^α^	43	Ha *et al.* (2000 ▸)
MurG–UDP–GlcNAc from *E. coli* (1nlm)	41.5	2.0 over 329 C^α^	43	Hu *et al.* (2003 ▸)
MGD1 from *A. thaliana* (4wyi)	23.7	3.3 over 285 C^α^	19	Rocha *et al.* (2016 ▸)
KCN28 from *Kitasatospora* (6j31)	23.3	3.6 over 297 C^α^	18	Shi *et al.* (2019 ▸)
